# Across Time: A Chronological Progression of Clinical Trials in India

**DOI:** 10.7759/cureus.56786

**Published:** 2024-03-23

**Authors:** Sohilkhan R Pathan, Vishal V Bhende, Kruti B Sharma, Vishal A Patel, Dinesh M Gangoda, Tanishq S Sharma

**Affiliations:** 1 Clinical Research Services, Bhanubhai and Madhuben Patel Cardiac Centre, Shree Krishna Hospital, Bhaikaka University, Karamsad, IND; 2 Pediatric Cardiac Surgery, Bhanubhai and Madhuben Patel Cardiac Centre, Shree Krishna Hospital, Bhaikaka University, Karamsad, IND; 3 Community Medicine, SAL Institute of Medical Sciences, Ahmedabad, IND

**Keywords:** regulatory framework, ethical guidelines, evolution, india, clinical research

## Abstract

The journey of clinical research in India spans centuries, marked by significant milestones and advancements in scientific, ethical, and regulatory domains. From early trials conducted by pioneers like James Lind to modern standards shaped by landmark events such as the Nuremberg Code and the adoption of Good Clinical Practice guidelines, India's progression reflects a commitment to ethical conduct and patient welfare. The Indian Council of Medical Research (ICMR) has played a pivotal role in this evolution, establishing national research centers and ethical committees to oversee biomedical research. Regulatory frameworks, exemplified by Schedule Y of the Drugs and Cosmetics Act, have adapted over time to align with global standards, facilitating India's integration into the international clinical development landscape. Despite challenges and setbacks, including misconceptions surrounding regulatory reforms, India's clinical trial ecosystem continues to evolve, driven by a dedication to ethical research practices and excellence in healthcare.

## Editorial

The journey of clinical research, from ancient trials to modern standards, marks a saga of scientific, ethical, and regulatory evolution. The key milestones include James Lind's scurvy trial in 1747, the first double-blind controlled trial by the UK Medical Research Council in 1943, and the groundbreaking streptomycin trial in 1946 [1-2]. The ethical framework for protecting human subjects in research traces back to ancient principles, notably the Hippocratic Oath's emphasis on avoiding harm. However, historical abuses, notably during World War II, spurred significant advancements in human subject protection. The Nuremberg Code of 1947 marked a pivotal moment, emphasizing the importance of voluntary informed consent in medical research [3]. Subsequent milestones, including the Universal Declaration of Human Rights in 1948 and the Kefauver-Harris amendments of 1962, strengthened oversight and consent requirements in research, especially in response to tragedies like the thalidomide incident [4-6]. The Helsinki Declaration of 1964 provided further guidelines, continually updated to address evolving ethical concerns [7]. However, issues like placebo use and post-trial access remain contentious. Legal instruments, such as the International Covenant on Civil and Political Rights, underscored the right to refuse medical treatment without consent, further reinforced by revelations of abuses like the Tuskegee study [8]. In the United States, the National Research Act of 1974 and the Belmont Report of 1979 were pivotal in shaping ethical standards for human research [9]. The adoption of Good Clinical Practice guidelines in 1996 further standardized ethical conduct in clinical trials on a global scale. Simultaneously, regulatory bodies like the FDA (Food and Drug Administration), established in 1862, evolved to enforce laws governing drug development and testing [10-11]. As science advances, the regulatory and ethical landscape will continue to adapt to new challenges and technologies, ensuring the protection and welfare of research participants.

The Indian Council of Medical Research (ICMR) has been pivotal in advancing medical research in India. From its inception in 1911 as the Indian Research Fund Association (IRFA), notable milestones include the initiation of medical research journal publication in 1912 and the establishment of the first Clinical Research Unit in 1945. In 1949, IRFA transformed into ICMR, marking an expanded focus. Over the following six decades, ICMR established numerous national research centers spanning various fields, cementing its role in shaping healthcare and scientific inquiry in India [12].

The establishment of the Central Ethical Committee of ICMR on Human Research, chaired by Hon'ble Justice (Retired) M.N. Venkatachaliah, marked a significant milestone in ethical oversight in India's biomedical research. Formed in 1996, this committee addressed specific ethical concerns through subcommittees focused on areas like epidemiological research, clinical evaluation of products, organ transplantation, and human genetics [13]. In 2000, the committee released the Ethical Guidelines for Biomedical Research, which were later revised in 2006. Parallel to these developments, Schedule Y of the Drugs and Cosmetics Act came into force in 1988, setting regulatory guidelines for the permission for clinical trials (CT). While it propelled growth in India's pharmaceutical industry, it also posed challenges by permitting only lower-phase trials, hindering global integration in clinical development. A vital shift occurred with the revision of Schedule Y in 2005, offering broader definitions for trial phases and removing restrictions on patient numbers and centers in early phases. This revision facilitated concurrent Phase II-III trials, thus aligning India with global standards. Moreover, the revision of Schedule Y in 2005 formalized Indian Good Clinical Practice (GCP) guidelines, outlining responsibilities for ethics committees, investigators, and sponsors. These amendments moved India towards GCP-compliant trials, providing crucial regulatory support for ethical and quality research practices [13-14].

In early 2013, the regulatory framework governing clinical research underwent a significant transformation, sparking widespread media attention and misconceptions about a supposed ban on such activities in India. While there was indeed a decrease in the number of ongoing and planned trials following this period, it was primarily due to a cautious approach adopted by sponsors who awaited clarification on the perceived stricter requirements imposed on conducting trials. These requirements included provisions for the examination of serious adverse events and protocols for compensating individuals in the event of a trial-related injury or fatality. Additionally, there was a mandate for audio-visual recording of the informed consent process, an imposition of limits on the number of trials an investigator could concurrently undertake, and a demand for a minimum number of public medical institutions meeting predefined standards to serve as trial sites. Furthermore, enhanced inspection and monitoring of trials, along with registration prerequisites for Ethics Committees, were introduced. While these reforms addressed longstanding concerns within the clinical research landscape, the absence of clear regulatory guidance on certain issues, ambiguity in legal terminology, and a lack of effective communication strategies from regulatory authorities resulted in confusion and uncertainty among stakeholders. This unintended consequence can be attributed to an "over-correction" in response to previous shortcomings. Compounding these challenges was the absence of a comprehensive resource detailing the nature of clinical trials conducted in India, leading to misconceptions and a skewed public discourse on the matter. Consequently, India's global standing in the realm of clinical research suffered a setback, as perceptions of regulatory stringency and uncertainty overshadowed the country's potential as a research destination. Moving forward, there is a pressing need for regulatory authorities to provide clarity, streamline processes, and enhance communication channels to rebuild trust and confidence in India's clinical research ecosystem. By fostering a transparent and supportive environment, India can reaffirm its position as a leading hub for ethical and high-quality clinical research, contributing to global advancements in healthcare while safeguarding the welfare of research participants [15].

There has been a rise and fall in clinical trials in India in the past two decades. The fall happened when the investigator and IRB (Institutional Review Board) failed to protect the trial participants, resulting in a revision of the Indian trial regulations. The new regulations were stringent and increased the time, effort, and cost, causing investigators to shy away from clinical trials [16]. Over the years, the landscape of clinical trials in India has undergone a remarkable transformation, marked by significant advancements, evolving regulations, and a growing emphasis on ethical conduct and patient welfare. From humble beginnings to becoming a key player in the global pharmaceutical arena, India's journey in clinical research is a testament to its resilience, adaptability, and unwavering commitment to excellence. In the early stages of its clinical trial journey, India emerged as an attractive destination for pharmaceutical companies seeking to capitalize on its large and diverse patient population, skilled medical professionals, and cost-effective research solutions [17]. However, alongside the opportunities came challenges, as regulatory frameworks were in their rising stages, and concerns regarding ethics and quality assurance loomed large. Despite the initial hurdles, India embarked on a path of continuous improvement and refinement. Regulatory authorities recognized the need for robust oversight and introduced measures to ensure the safety and well-being of trial participants. Guidelines were strengthened, and mechanisms for ethical review and approval were put in place to uphold the highest standards of integrity and transparency.

As India's clinical trial ecosystem has matured, so has its infrastructure and expertise, marking a significant evolution in the landscape of medical research within the country. This transformation has been supported by substantial investments in healthcare infrastructure, the establishment of state-of-the-art research facilities, and the encouragement of human capital, all of which have contributed to enhancing India's capacity to conduct trials up to international standards. Crucially, collaborations between academia, industry, and government agencies have been instrumental in fostering a culture of innovation and knowledge exchange. These partnerships have facilitated the sharing of resources, expertise, and best practices, ultimately positioning India as a hub for cutting-edge research and development in the global arena.

Technological advancements have played a pivotal role in revolutionizing the conduct of clinical trials within India. The adoption of electronic data capture systems, telemedicine platforms, and other digital tools has significantly enhanced the efficiency, accuracy, and reliability of trial processes. These innovations have streamlined data collection, monitoring, and analysis, while also reducing administrative burdens and improving overall trial management. Amidst these advancements, a growing emphasis on ethical considerations and patient-centricity has emerged as a guiding principle in the evolution of clinical trials in India. Stakeholders across the spectrum, including researchers, regulators, sponsors, and advocacy groups, have increasingly prioritized the protection of participants' rights and welfare. This commitment has been reflected in efforts to ensure informed consent, promote community engagement, and uphold the highest ethical standards throughout the research process.

**Figure 1:**
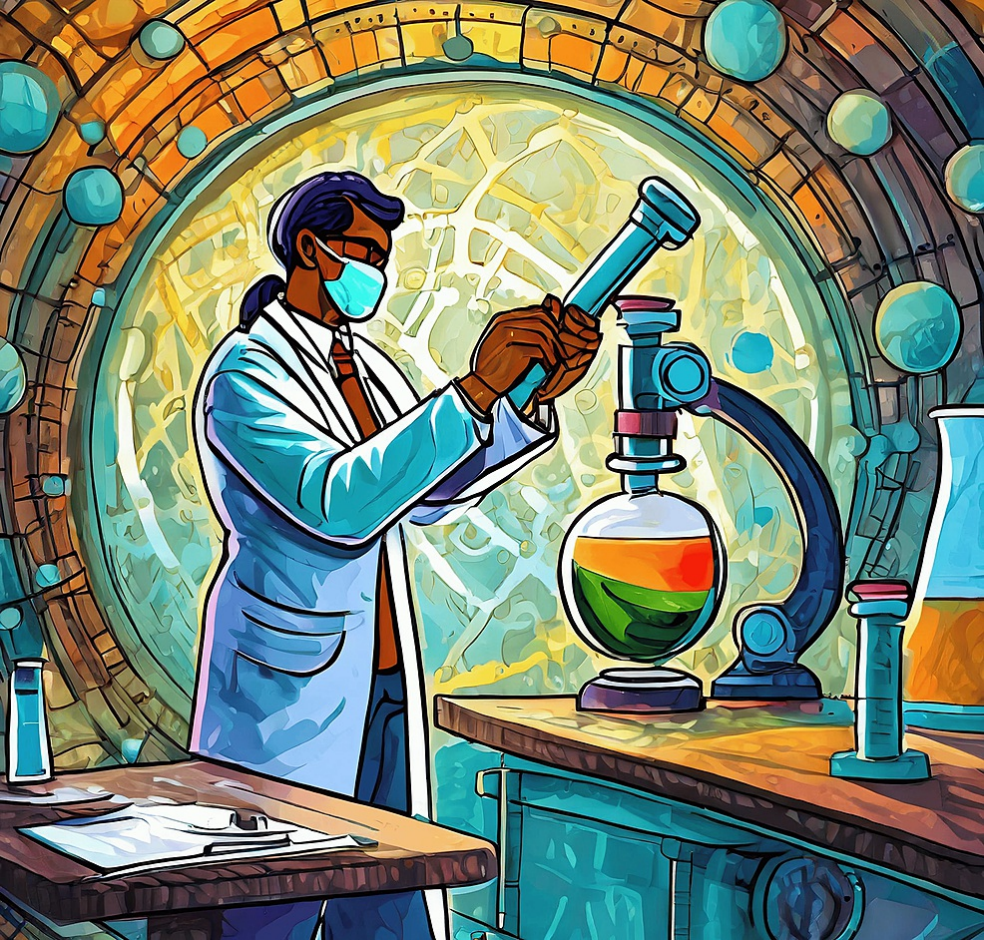
The image was created by Sohilkhan R. Pathan on February 19, 2024, using OpenAI's AI-Generated Image Exploring [Adobe]. Retrieved from https://www.adobe.com/products/firefly/features/text-to-image.html.

Today, as India emerges as a key player in the domain of global clinical research, it becomes increasingly important to recognize both, the steps made and the hurdles that remain. While significant progress has undoubtedly been achieved, there are still critical areas that demand attention and determined efforts. One such area is the need to enhance access to healthcare services, particularly for marginalized and underserved communities. Ensuring equitable access to clinical trials not only promotes inclusivity but also facilitates the development of interventions that address the diverse needs of the population. Additionally, addressing disparities in research participation is essential for the integrity and effectiveness of clinical trials. Efforts to increase diversity among study participants, including better representation of different demographic groups, can lead to more strong and generalizable findings. Furthermore, there is a pressing need to strengthen post-trial follow-up and pharmacovigilance mechanisms to monitor the long-term safety and efficacy of medical interventions. Proactive surveillance and monitoring systems can help identify and reduce potential risks associated with new treatments, thereby safeguarding public health. As we reflect on India's journey in clinical trials, it is clear that the path forward holds huge promise and potential. By continuing to invest in healthcare infrastructure, fostering cross-sector collaborations, embracing technological innovations, and upholding the highest ethical standards, India can further solidify its position as a global leader in clinical research. Through these determined efforts, India can not only drive medical advancements but also contribute to improving health outcomes for populations worldwide. As we navigate the complexities of modern healthcare, India's continued commitment to excellence in clinical research will undoubtedly shape the future of medicine and surface the way for a healthier, more equitable world (Figure 1).

Conclusion

In conclusion, the evolution of clinical trials in India is a story of resilience, innovation, and continuous improvement. From its humble beginnings to its current position as a global leader in clinical research, India has overcome numerous challenges and embraced opportunities for growth and development. The journey has been marked by advancements in regulatory frameworks, infrastructure, technology, and a steadfast commitment to ethical conduct and patient welfare. As we look towards the future, it is essential to build upon the progress made and address remaining challenges with determination and collaboration. By fostering a supportive ecosystem that encourages innovation, promotes transparency, and prioritizes the well-being of participants, India can further enhance its contribution to global healthcare advancements. Ultimately, the evolution of clinical trials in India is not just a testament to the country's scientific excellence but also its unwavering dedication to improving the lives of people worldwide. As we embark on the next phase of this journey, let us remain guided by the principles of integrity, inclusivity, and excellence, ensuring that the promise of clinical research is realized for the benefit of all.

## References

[1] Tröhler U (2005). Lind and scurvy: 1747 to 1795. J R Soc Med.

[2] Crofton J (2006). The MRC randomized trial of streptomycin and its legacy: a view from the clinical front line. J R Soc Med.

[3] Ghooi RB (2011). The Nuremberg Code-a critique. Perspect Clin Res.

[4] Gostin LO, Meier BM, Thomas R, Magar V, Ghebreyesus TA (2019). 70 years of human rights in global health: drawing on a contentious past to secure a hopeful future. Lancet.

[5] Hollister LE, Page IH, Pfeiffer CC, Visscher MB (1968). The Kefauver-Harris amendments of 1962: a critical appraisal of the first five years. J Clin Pharmacol J New Drugs.

[6] Kim JH, Scialli AR (2011). Thalidomide: the tragedy of birth defects and the effective treatment of disease. Toxicol Sci.

[7] Goodyear MD, Krleza-Jeric K, Lemmens T (2007). The Declaration of Helsinki. BMJ.

[8] Levine RS, Williams JC, Kilbourne BA, Juarez PD (2012). Tuskegee redux: evolution of legal mandates for human experimentation. J Health Care Poor Underserved.

[9] Department of Health, Education Education, and Welfare; National Commission for the Protection of Human Subjects of Biomedical and Behavioral Research (2014). The Belmont Report. Ethical principles and guidelines for the protection of human subjects of research. J Am Coll Dent.

[10] Vijayananthan A, Nawawi O (2008). The importance of Good Clinical Practice guidelines and its role in clinical trials. Biomed Imaging Interv J.

[11] Wang W, Wertheimer AI (2022). History, status, and politicization of the FDA. Res Social Adm Pharm.

[12] (2024). Indian Council of Medical Research. https://main.icmr.nic.in/.

[13] (2024). National Ethical Guidelines for Biomedical and Research Involving Human Participants. https://ethics.ncdirindia.org/asset/pdf/ICMR_National_Ethical_Guidelines.pdf.

[14] Ministry of Health and Familiy Welfare (Department of Health) (2016). The Drugs and Cosmetics Act and rules. https://cdsco.gov.in/opencms/export/sites/CDSCO_WEB/Pdf-documents/acts_rules/2016DrugsandCosmeticsAct1940Rules1945.pdf.

[15] (2024). Challenges and prospects for clinical trials in India - a regulatory perspective. https://icrier.org/pdf/Challenges-and-prospects-of-clinical-trials-in-India.pdf..

[16] Mallath MK, Chawla T (2017). Investigators' viewpoint of clinical trials in India: past, present and future. Perspect Clin Res.

[17] Poongothai S, Unnikrishnan R, Balasubramanian J, Nair MD, Mohan V (2014). Why are clinical trials necessary in India?. Perspect Clin Res.

